# Support-Seeking Strategies and Social Support Provided in Chinese Online Health Communities Related to COVID-19

**DOI:** 10.3389/fpsyg.2021.783135

**Published:** 2021-11-17

**Authors:** Yi Liu, Yiwei Zhu, Yixuan Xia

**Affiliations:** School of Journalism and Communication, Chongqing University, Chongqing, China

**Keywords:** support-seeking strategies, social support, Chinese online health communities, COVID-19, content analysis

## Abstract

Online health communities have become one of the most important means for people to seek social support during the coronavirus 2019 disease (COVID-19) pandemic. This study details content analysis of support-seeking strategies and social support offered on the online forum “Baidu COVID-19bar” across different stages of initial stage as well as during the entire initial stage of the COVID-19 pandemic. The results show that asking for support and disclosing directly were the main strategies used across the different stages and during the entire initial stage. Informational support and emotional support were the most common types sought in the first two stages and the entire initial stage, and informational support was the main type during the decline stage. Furthermore, asking for support was more likely to elicit informational support while disclosing directly was more likely to elicit emotional support. Theoretical and practical implications of the findings are discussed.

## Introduction

In January 2020, Coronavirus Disease 2019 (COVID-19) broke out. The COVID-19 pandemic is a major public health emergency, having demonstrated a rapid transmission speed, wider infection range, and is the hardest to control of any disease in recent years. It has been listed as a “Public Health Emergency of International Concern” by the World Health Organization (WHO, 2020). Especially, in the initial stage of the COVID-19 pandemic (from January 2020 when the epidemic began to spread to March when the number of new confirmed cases per day on the mainland was kept within single digits) (The State Council Information Office of the People’s Republic of China, 2020), people were asked to execute home quarantine to prevent the spread of the pandemic, which caused anxiety and inadequate understanding of information. Therefore, people tended to seek information to reduce the risk of uncertainty (Xu et al., 2020; Tanis, 2008).

With the popularization of mobile terminal equipment and the development of network communication technology, people can search for and discuss information about the COVID-19 in online communities. Online communities, through collaborative technologies and the characteristics of being quick to use, interactive, and low-cost, enable users to communicate, coordinate, and collaborate to promote group interaction, and in turn build and share information (Panteli and Sims, 2010; Sims, 2011).

In addition, research by Huh (2015) has suggested that because of the anonymity afforded by online communities, some users may be willing to share private topics and stories, and engage in discussions with people who have similar issues for seeking or providing social support. Social support is a form of social connection, and it is considered to be the support obtained by individuals through establishing social connections with other individuals, groups, and larger communities (Lin et al., 1979; Wang et al., 2019). Therefore, online health communities have become valuable sources for users to seek what they need or provide social support during the initial period of COVID-19.

In previous studies related to the COVID-19 epidemic, few studies have focused on the relationship between social support-seeking strategies and social support. When it comes to social support, previous studies pay more attention to analyse under the background of individualist culture. To fill the gap, this study is conducted against the background of collectivism in China, and analyzes the types and relevance of social support-seeking strategies and support provided in COVID-19. Also, few previous studies have explored the social support during different stages of COVID-19. Therefore, based on the life cycle of the crisis and the development trends of the epidemic, this study divides the initial period of the COVID-19 pandemic into three stages and analyses whether there are differences about support-seeking strategies and social support provided, as well as the relationship between the two.

## Literature Review

### Stages of COVID-19

Research on crisis and risk communication has posited that crisis events have the characteristics of importance, immediacy, and uncertainty (Dutton, 1986), which vary at different stages of the process, from its emergence to its end, also called it “life cycle” (Fink, 1986). The life cycle of a crisis usually goes through a stage of accumulation of potential threats before the crisis, to a stage in which various triggering events cause the crisis to break out, and then to a stage in which the crisis slowly decreases and ends, and the impact gradually weakens.

**Figure 1:**
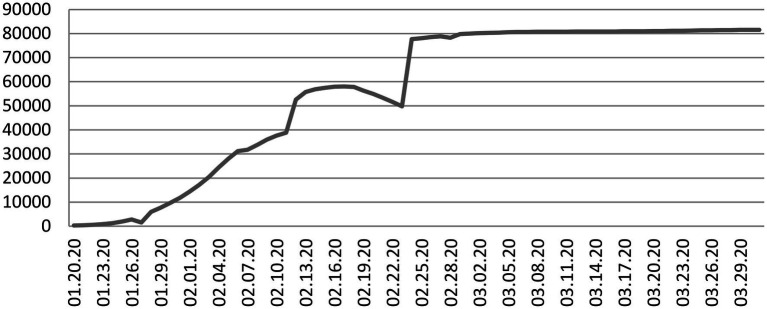
Trajectory of confirmed cases of COVID-19 in China, 01.20.20–03.31.20.

**Figure 2:**
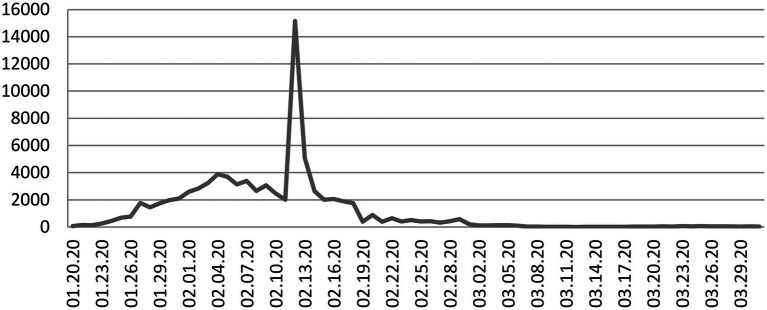
Trajectory of daily confirmed cases of COVID-19 in China, 01.20.20–03.31.20.

As a global public health emergency, COVID-19 also followed a particular development order in China during the initial period. According to the daily confirmed cases and latest confirmed cases released by the National Health Commission of the People’s Republic of China, certain trajectories were followed. As shown in Figures 1 and 2, from January 20 to the end of January, although the country’s daily cases were beginning to increase rapidly, the number of confirmed cases was small. However, COVID-19 was gradually spreading, and various crisis factors continued to accumulate; from early February to mid-February, the number of confirmed cases increased rapidly, and the number of daily new infections peaked. At this stage, people’s uncertainty increased. From mid-February to late March, the number of daily confirmed cases dropped rapidly, and the number of new cases was effectively controlled, and the negative effects gradually decreased. Based on the above, this study divides the initial development of COVID-19 into three stages: the early stage (January 20 to the end of January), the peak stage (early to mid-February 2020), and the decline stage (mid-February 2020 to the end of March 2020).

### Social Support-Seeking Strategies

Seeking support is defined as “intentional communicative activity with the aim of eliciting supportive actions from others” (MacGeorge et al., 2011, p. 330). Barbee and Cunningham (1995) proposed the sensitive interaction systems theory (SIST) to explain how people seek support which includes several dimensions of support-seeking strategies: direct/indirect and verbal/nonverbal. However, with the rapid development of mobile Internet technology and new media, online communities have become one of the main channels for people to send requests for help and exchange views and opinions with others, which means that SIST applied in a face-to-face communication scenario is no longer a sufficient explanation of how people seek social support. On the one hand, nonverbal strategies (e.g., crying and grieving) cannot be applied to online communities, while verbal strategies (e.g., discussing problems and complaining) cannot cover all forms of verbal expression. In online communities, users tend to diversify their expressions used, and can convey their willingness to seek social support through texts, pictures, videos, and even emojis; thus, verbal strategies relying on oral expression cannot explain such behavior. On the other hand, SIST argues that direct seeking strategies are explicit—that is, people articulate their problems in detail and make their needs known directly—while indirect seeking strategies refer to relatively unclear ways of expressing needs, such as complaining and changing the subject. Indeed, online communities provide people with more time and energy to devise seeking strategies compared to face-to-face communication. Complex seeking strategies can potentially include both direct and indirect forms, and can even emerge with other dimensions. To overcome this gap, Buehler et al. (2019) conducted a thematic analysis of text data on Facebook and proposed seven types of seeking strategies: asking for support, disclosing directly, alluding to the stressor, using humor, celebrating achievements, sharing stories and memories, and shouting out. These strategies expand on SIST and reveal that language strategies in social media are not limited to the direct and indirect categories. People are beginning to devise a variety of strategies to seek the social support of others *via* visible and persistent communication channels.

The raging COVID-19 pandemic has been a great source of anxiety, distress, and stress among the population (Kanekar and Sharma, 2020). Because of the uncertainty caused by COVID-19, people have found it difficult to obtain accurate information about the symptoms, effects, and treatment of the disease. At the same time, compulsory control measures have restricted people’s social interaction activities and further increased their anxiety and depression. When people buffer stress, they often choose to seek help to cope with troubles or stressors (Goldsmith, 2004). Their distress affects their communication with relatives, friends, and other important people, as well as affecting the exchange of social support information in turn. It should be pointed out that the negative psychological effects of the pandemic were not static, and varied across the different stages. Studies have shown that as the number of new cases and deaths rose, the anxiety level of college students also increased (Kleiman et al., 2020). Likewise, during the decline stage, the anxiety of lonely people reduced accordingly (Xu et al., 2020). Therefore, the different psychological conditions of individuals at different stages of the pandemic will have affected their needs for social support accordingly, causing them to pursue different seeking strategies.

In order to observe the individual support-seeking strategies in social media from a new and holistic perspective, the following research questions (RQs) are proposed:

RQ1a: What types of social support-seeking strategies were used in health communities across different stages of the initial development of COVID-19?
RQ1b: What types of social support-seeking strategies were used in health communities during the entire initial stage?

### Social Support

Providing social support refers to offering and expressing support to others. This behavior not only enables others to receive care and satisfaction but also has a positive psychological effect on the provider (Bokszczanin, 2012). Specifically, providing social support may enhance the provider’s sense of competence and confidence, as well as improving their subjective well-being. Experiences such as positive emotions, the feeling of being needed and highly valued by others, increased social contact, and more in-depth contact with others produces positive psychological results (Bokszczanin, 2012; Ye et al., 2019). Especially in disaster events, the behavior of helping others can protect individuals from feelings of helplessness and inadequacy, and allow them to regain a sense of control and self-efficacy when they suffer negative effects of the disaster (Bokszczanin, 2012). Therefore, when faced with the emergency comprising COVID-19, individuals were willing to provide social support in order to gain positive feelings from helping others through answering their questions and giving them comfort.

Providing social support information through the Internet has gradually become a common phenomenon. People can take more time and energy, compared to in face-to-face scenarios, to think about how to edit and organize complex social support-seeking information (Oh and LaRose, 2016) with the help of multiple media options such as text and pictures. Cutrona and Suhr’s (1992) classification of social support, which has been widely used in content analysis of online communities, includes five categories: (1) informational support: advice, referral, situation appraisal, and teaching; (2) emotional support: relationship, physical affection, sympathy, empathy, encouragement, and prayer; (3) esteem support: compliment, validation and relief of blame; (4) network support: access, presence, and companionship; (5) tangible support: lending something (such as money), performing a task directly related to the stress, expressing willingness to help, etc. Previous studies have found that the main types of social support in online communities are different. For example, informational support and emotional support are the most common types in online self-help groups for anxiety and depression (Yip, 2020); in HIV communities, informational support and emotional support are the most common, while esteem support and network support are less important (Maestre et al., 2018).

Thus, the following research questions are proposed:

RQ2a: What types of social supports were offered in health communities across different stages of the initial development of COVID-19?
RQ2b: What types of social supports were offered in health communities during the entire initial stage?

### Support-Seeking Strategies and Social Support

Whether and how social network members provide support is at least partially contingent on the strategies employed by individuals to ask for help (Barbee and Cunningham, 1995). Seeking and providing social support is a process of exchanging of information. Seekers usually describe their own experiences and emotions to the provider and convey their desire for social support. After obtaining relevant information, the provider assesses it and provides support to the seeker accordingly. Within this process, the clarity of the seeker’s information and the degree of indication of the need for social support affect the provider’s willingness and ability to provide support. According to SIST, in the direct support-seeking strategy, people usually describe their problems in detail and express what they need, thus enabling others to understand how to provide support. Therefore, the direct support-seeking strategy is more likely to result in the receipt of social support (Williams and Mickelson, 2008), and in approach (e.g., solve) responses (Barbee and Cunningham, 1995). In the indirect support-seeking strategy, vague expression of needs may cause the provider to lack understanding of how to provide social support and of whether they can provide support explicitly (Barbee et al., 1998); thus, an indirect support-seeking strategy is more likely to result in less social support and in avoidance (e.g., dismiss) responses (Barbee and Cunningham, 1995).

The phenomenon of people asking for help, followed by other people observing these posts and trying to provide support, is common in online communities centered on COVID-19. Although previous studies have examined the relationship between direct/indirect strategies and social support, people spend significant time and energy applying a variety of strategies to satisfy the need for support in online communities; thus, in other words, the two dimensions (direct/indirect) cannot fully account for people’s use of strategies, nor for whether and how other strategies are used to elicit support. Therefore, in order to study the mechanisms underlying the seeking and giving of support during COVID-19, we introduce Buehler et al.’s (2019) seven strategies and examine whether these strategies result in social support, and, if so, which type they elicit.

Different characteristics that emerged across the different stages of the initial COVID-19 outbreak may have influenced the relationship between strategies and support. To assess the relationship, therefore, the following research questions are proposed:

RQ3a: What is the relationship between support-seeking strategies and social support offered in health communities across different stages of the initial development of COVID-19?
RQ3b: What is the relationship between support-seeking strategies and social support offered in health communities during the entire initial stage?

## Materials and Methods

### Data Sources and Sample

In order to generate a representative sample, this study collected data from Baidu Tieba, which is a widely used Chinese communication platform likened to the popular American classified ads website Craigslist (Lee, 2021). It provides a platform for people to make comments with the characteristics of large scale, high maturity, convenient user communication, and structured display of information. In Baidu Tieba, users’ postings and responses directly or indirectly manifest different types of social support provided and social support-seeking strategies (Qian et al., 2021). What’s more, Baidu Tieba established “Baidu COVID-19bar” to provide a platform for people who have encountered similar difficulties about COVID-19 on January 20, 2020. By March 5, 2021, “Baidu COVID-19bar” had 221, 412 followers and 1,533,574 posts which has large amounts of users and posts, and the level of interaction is frequent (Baidu COVID-19bar, 2020). The data contained therein are centralized and convenient to obtain for the analysis of online social support (Yao et al., 2021). Therefore, we use the “Baidu COVID-19bar” as the data source for our content analysis.

Baidu Tieba provides a network platform for people to discuss on where data are freely accessible to everyone and convenient to collect. In order to avoid violating the privacy of users, we use numbers instead of usernames to refer to posters in order to maintain their anonymity. We used Python to crawl the contents of the “Baidu COVID-19bar,” including the content of each post and of replies thereto, from January 20, 2020 to the March 31, 2020. After screening out repetitive posts and posts that did not contain social support-seeking strategies, the number of posts for each stage was as follows: Stage 1: 199 original posts and 8,730 replies; Stage 2: 257 original posts and 7,492 replies; Stage 3: 355 original posts and 2,373 replies. The number of posts in the first stage was less than those in the second and third stages, but the number of replies during that stage was the highest. In the third stage, the number of posts was highest but the number of replies was much lower than in the other two stages. A total of 600 sample posts and corresponding replies were selected by random number generators from each stage and the overall three-stage sample to explore whether there were differences between each stage and how each stage compared with the overall situation.

### Coding of Original Posts and Replies

We coded the original posts in the three stages based on the seven types of social support-seeking strategies proposed by Buehler et al. (2019), including asking for support, disclosing directly, alluding to the stressor, using humor, celebrating achievements, sharing stories and memories, and shouting out.

Asking for support refers to an individual’s need for information, advice, prayers, material help, ideas, and so on. Disclosing directly refers to the individual attracting the attention of others by expressing their feelings and experiences, which are related to their stressors, while not directly stating their need for support. Alluding to the stressor entails not directly pointing out the stressor; instead, individuals often imply the stressor through vague expressions, rhetoric, and citations. Using humor entails referring to something in a way that is intended to be funny. Celebrating achievements refers to an individual’s elaboration of an achievement, such as finding a job or recovering from an illness. Sharing stories and memories includes posting regrets about the loss of loved ones and detailing events that happened in the past. Shouting out refers to expressing appreciation for what a person or group is doing for an individual during a difficult period. Because the seven types of strategies differ in the degree of directness and indirectness, they cannot be simply classified as direct or indirect strategies. Thus, the above coding method entailed subdividing the complex support-seeking strategies in the context of social media into multi-dimensional and multi-level segments.

Based on the Social Support Behavior Code, we coded all replies except those from the original post publisher, including informational support, emotional support, esteem support, network support, and tangible support.

Informational support refers to providing advice or solutions to help the poster reanalyze and evaluate their problem from another perspective, as well as providing detailed facts and news. Emotional support refers to the expression of regret and sympathy for the problems or situations faced by the poster, and the detailing of similar experiences to convey understanding, as well as hope and encouragement, to the poster. Esteem support refers to expressing praise for the views or behaviors of the poster, affirming the views or behaviors of the poster, comforting the poster to assuage their feelings of guilt, and so on. Network support refers to providing a sense of belonging to others by expressing similar experiences. Tangible support refers to conveying a willingness to help the poster and providing substantial help to the poster.

Based on the above operational definitions of seeking strategy and social support, the identifications of variable types were constructed. The seven types of support-seeking strategies and five types of social support identified above were coded as “present” (1) or “not present” (0) on the posts considered.

All coding was carried out by two coders who completed in-depth training on the topic. The reliability test is completed by measuring inter-coder agreement and by calculating the reliability using the reliability test formula, which is as follows:

1. inter-coder agreement: k=2 * M/ (N1+N2)
2. reliability: r(reliability)=n * k/[1+(n − 1) * k]

M refers to the number of times coders had the same outcome; N1 refers to the number of times the first coder coded; N2 refers to the number of times the second coder coded; n refers to the number of coders; k refers to the inter-coder agreement.

As shown in Table 1, the reliability of all variable types was greater than 0.90.

**Table 1:** Inter-coder agreement and reliability of social support-seeking strategies and social support provided.

Variables	Inter-coder agreement	Reliability
**Social support-seeking strategies**
Asking for support	0.90	0.95
Disclosing directly	0.87	0.92
Alluding to the stressor	0.90	0.95
Using humor	1.00	1.00
Celebrating achievements	1.00	1.00
Sharing stories and memories	1.00	1.00
Shouting out	0.97	0.98
**Social support**
Informational support	0.90	0.95
Emotional support	0.93	0.97
Esteem support	0.93	0.97
Network support	0.97	0.98
Tangible support	1.00	1.00

## Results

### Support-Seeking Strategies

RQ1a and RQ1b asked what types of support-seeking strategies were used in the health community across different stages and during the initial period. To answer RQ1a and RQ1b, we conducted frequency analysis to examine the support-seeking strategies in the original posts. As shown in Table 2, in the early stage asking for support and disclosing directly were the most common types; alluding to the stressor and shouting out were relatively less frequent; and using humor, sharing stories and memories, and celebrating achievements were not observed, in the peak stage, asking for support and disclosing directly were still the main types, followed by shouting out and alluding to the stressor. Using humor, sharing stories and memories, and celebrating achievements were not observed. Similarly, in the decline stage, asking for support and disclosing directly were the most common types and the other five strategies were not observed. Therefore, people mainly adopted asking for support and disclosing directly across the three stages, and these two strategies were used much more frequently compared to others. This result is consistent with findings across the entire stage. In addition, asking for support was used more often than disclosing directly.

**Table 2:** Support-seeking strategies across the three stages.

Support-seeking strategies	Frequency
**Early stage**	
Asking for support	390 (65.00%)
Disclosing directly	165 (27.50%)
Alluding to the stressor	21 (3.50%)
Using humor	0 (0.00%)
Celebrating achievements	0 (0.00%)
Sharing stories and memories	0 (0.00%)
Shouting out	21 (3.50%)
**Peak stage**	
Asking for support	377 (62.80%)
Disclosing directly	220 (36.70%)
Alluding to the stressor	2 (0.30%)
Using humor	0 (0.00%)
Celebrating achievements	0 (0.00%)
Sharing stories and memories	0 (0.00%)
Shouting out	49 (8.20%)
**Decline stage**	
Asking for support	517 (86.20%)
Disclosing directly	218 (36.30%)
Alluding to the stressor	0 (0.00%)
Using humor	0 (0.00%)
Celebrating achievements	0 (0.00%)
Sharing stories and memories	0 (0.00%)
Shouting out	0 (0.00%)
**All stages**	
Asking for support	413 (68.80%)
Disclosing directly	301 (50.20%)
Alluding to the stressor	0 (0.00%)
Using humor	1 (0.20%)
Celebrating achievements	0 (0.00%)
Sharing stories and memories	0 (0.00%)
Shouting out	14 (2.30%)

To sum up, the use of social support-seeking strategies across the three stages was consistent with the entire stage. Asking for support and disclosing directly were the main types, and asking for support was used more frequently compared to disclosing directly. Alluding to the stressor, shouting out, using humor, sharing stories and memories, and celebrating achievements were used least often.

### Social Support

RQ2a and RQ2b asked what types of social support were offered in the health community across different stages and during the entire stage. To answer RQ2a and RQ2b, we conducted frequency analysis to examine the social support offered in the reply posts. As shown in Table 3, in the early stage, informational support and emotional support were the most common types followed by esteem support, network support, and tangible support. Similar to the early stage, informational support and emotional support were the most common types during the peak stage, followed by esteem support, network support, and tangible support. In the decline stage, although informational support was the main type, the amount of emotional support clearly decreased. The amount of network support and tangible support are still less than the amount of emotional support, and esteem support was not observed. During the entire stage, informational support and emotional support were the most common types, with other types being used much less frequently.

**Table 3:** Social support across the three stages.

Support type	Frequency
**Early stage**	
Informational support	306 (51.00%)
Emotional support	141 (23.50%)
Esteem support	17 (2.80%)
Network support	1 (0.20%)
Tangible support	4 (0.70%)
**Peak stage**	
Informational support	277 (46.20%)
Emotional support	153 (25.50%)
Esteem support	14 (2.30%)
Network support	5 (0.80%)
Tangible support	3 (0.50%)
**Decline stage**	
Informational support	391 (65.20%)
Emotional support	7 (1.20%)
Esteem support	0 (0.00%)
Network support	1 (0.20%)
Tangible support	4 (0.70%)
**All stages**	
Informational support	248 (41.30%)
Emotional support	72 (12.00%)
Esteem support	2 (0.30%)
Network support	3 (0.50%)
Tangible support	10 (1.70%)

To sum up, the results shown with respect to the early stage and the peak stage are similar to those for the entire stage. That is, people primarily offered informational support and emotional support. In this process, informational support was used more than was emotional support, and esteem support, network support, and tangible support occurred much less often. However, in the decline stage, although informational support was still the most common type, emotional support clearly decreased.

### Support-Seeking Strategies and the Social Support Provided

RQ3a and RQ3b asked about the relationship between support-seeking strategies and social support offered in health communities across different stages and the entire stages. To answer these questions, chi-square tests were conducted.

As shown in Table 4, in the early stage, seekers who used asking for support were more likely to elicit informational support than seekers who did not (*χ^2^*=21.53, *p*<0.001). On the contrary, seekers who did not use asking for support were more likely to elicit emotional support (*χ*^2^=57.77, *p*<0.001). Seekers who used disclosing directly were more likely to elicit emotional support than seekers who did not (*χ^2^*=67.94, *p*<0.001). In contract, seekers who did not use disclosing directly were more likely to elicit informational support than seekers who did (*χ*^2^=8.73, *p*<0.01).

**Table 4:** Results of chi-square test of asking for support, disclosing directly and social support in the early stage.

	Informational support		Emotional support	
Asking for support	YES	NO	YES	NO
YES	**226** ^***^	164	54	336
NO	80	130	**87** ^***^	123
Disclosing directly				
YES	68	97	**77** ^***^	88
NO	**238** ^**^	197	64	371

*** *p<0.001*,

** *p<0.01*.

As shown in Table 5, in the peak stage, seekers who used asking for support were more likely to elicit informational support than seekers who did not (*χ^2^*=17.88, *p*<0.001). On the contrary, seekers who did not use asking for support were more likely to elicit emotional support (*χ*^2^=34.12, *p*<0.001). Seekers who used disclosing directly were more likely to elicit emotional support than seekers who did not (*χ^2^*=31.55, *p*<0.001). In contrast, seekers who did not use disclosing directly were more likely to elicit informational support than seekers who did (*χ^2^*=16.04, *p*<0.001).

**Table 5:** Results of chi-square test of asking for support, disclosing directly and social support in the peak stage.

	Informational support		Emotional support	
Asking for support	YES	NO	YES	NO
YES	**199** ^***^	178	66	311
NO	78	145	**87** ^***^	136
Disclosing directly				
YES	78	142	**85** ^***^	135
NO	**199** ^***^	181	68	312

*** *p<0.001*.

As shown in Table 6, in the decline stage, seekers who used asking for support were more likely to elicit informational support than seekers who did not (*χ^2^*=27.39, *p*<0.001). Seekers who did not use disclosing directly were more likely to elicit informational support than seekers who did (*χ^2^*=14.08, *p*<0.001). Because expectation value is less than 5, the conclusion about asking for support, disclosing directly and emotional support cannot be drawn upon.

**Table 6:** Results of chi-square test of asking for support, disclosing directly and social support in the decline stage.

	Informational support	
Asking for support	YES	NO
YES	**358** ^***^	159
NO	33	50
Disclosing directly		
YES	121	97
NO	**270** ^***^	112

*** *p<0.001*.

As shown in Table 7, across the three stages, seekers who used asking for support were more likely to elicit informational support than seekers who did not (*χ^2^*=46.98, *p*<0.001). On the contrary, seekers who did not use asking for support were more likely to elicit emotional support (*χ^2^*=109.39, *p*<0.001). Seekers who used disclosing directly were more likely to elicit emotional support than seekers who did not (*χ^2^*=72.47, *p*<0.001). On the contrary, seekers who did not use disclosing directly were more likely to elicit informational support compared to seekers who did (*χ^2^*=8.34, *p*<0.01).

**Table 7:** Results of chi-square test of asking for support, disclosing directly, and social support during the three stages.

	Informational support		Emotional support	
Asking for support	YES	NO	YES	NO
YES	**209** ^***^	204	11	402
NO	39	148	**61** ^***^	126
Disclosing directly				
YES	107	194	70***	231
NO	**141** ^**^	158	2	297

*** *p<0.001*,

** *p<0.01*.

From the comparison of the three stages, it can be seen that the early stage is consistent with the peak stage—that is, asking for support was more likely to elicit informational support, while disclosing directly was more likely to elicit emotional support. In the decline stage, similarly, asking for support was more likely to elicit informational support. To sum up, the relationship between asking for support and informational support across the three stages was consistent with the entire stage.

## Discussion

From a general perspective, the “Baidu COVID-19bar” members mainly adopted strategies of asking for support and disclosing directly. Moreover, in the three stages, asking for support and disclosing directly were the main seeking strategies used.

There is a strong possibility that the individuals did not clearly understand information related to the pandemic and were worried about their situation, which may have resulted in anxiety, panic, and other emotions. In addition, support for information and advice, which can help people cope with uncertainty and reduce stress, can have a positive impact on individuals’ health outcomes (Ford et al., 1996; Brashers et al., 2004). Therefore, in order to mitigate the anxiety caused by the pandemic and understand information related to their safety, members tended to post their own questions to seek answers and lessen some of the uncertainty surrounding the pandemic situation; this strategy is known as asking for support.

In addition to seeking information and advice by asking questions on the “Baidu COVID-19bar,” users expressed their emotional needs by detailing their feelings and their experiences rather than directly explaining their information needs, so as to seek spiritual and emotional comfort; this is the strategy of disclosing directly. There is a strong possibility that the perceived social support during the pandemic provided users with a sense of companionship and belonging, as well as helping them reduce their long-term anxiety and enhancing their self-efficacy in coping with future uncertainty (Hawkley and Cacioppo, 2010; Taylor and Broffman, 2011; Xu et al., 2020). In addition, when users apply this strategy they confront the source of their distress directly, without necessarily verbally expressing their need for support, and thus do not explicitly express their feelings or the potential source of distress (Buehler et al., 2019).

In the analysis of the three stages and the entire stage, respectively, asking for support and disclosing directly were found to be the main seeking strategies. This may be because people’s uncertainty and insecurity were persistent during the pandemic, wherein people were seeking information about outbreaks, medicines, and treatments. Thus, social support can adjust individual emotions and behaviors by managing uncertainty and alleviating anxiety. Fewer users applied the other five strategies to seek social support, perhaps because the contents of these strategies are usually accompanied by other topics, especially asking for support and disclosing directly. In addition, users primarily directly sought informational support or expressed emotional needs to others in the “Baidu COVID-19bar.”

This study found that, on the whole, users of the “Baidu COVID-19bar” mainly provided informational support and emotional support. In the three stages, informational and emotional support were the main support-seeking strategies used. This finding is in line with those of previous social support studies, possibly because, compared with the traditional social support environment, online health communities have the characteristics of interactivity, anonymity, and stronger interactivity, and the content published by individuals can be seen by more people. These factors may make people more willing to publish information and provide support to others in the community (Wright et al., 2021). Perhaps because key aspects of online health community discussions include sharing experiences on diseases and treatment options (Nambisan, 2011), and because individuals interacting in a specific topic area have the same interests or face similar problems or situations, users tend to share their experiences and provide suggestions or references on similar issues (Ko et al., 2013). In addition, the emotion-related contents in the “Baidu COVID-19bar” may struck a chord with users who have gone through the same experience and inspired them to provide emotional help to others. In the context of collectivism, seekers can sense a great deal of hidden emotional support from providers from just a few sentences (Ko et al., 2013; Qian et al., 2021). In other words, in order to provide informational support to others through other types of social support strategies may require the user’s knowledge, understanding and organization of relevant information, while providing emotional support is simpler and only requires the expression of empathy. These factors may explain why emotional support is the primary support-seeking strategy. The fact that informational support and emotional support were the main social support strategies used may also be associated with the background of COVID-19, since during the outbreak of the pandemic, users were looking for more information; their emotional upset and anxiety may have prompted them to seek support, and their request for social support was often informational and emotional, so as to elicit more informational and emotional aspects of social support. Members of the “Baidu COVID-19bar” provided much less esteem support, network support, and tangible support. According to Hether et al. (2016), from the perspective of social network structure, the networks in the “Baidu COVID-19bar” exhibit weak ties and have the primary function of exchanging informational support; thus, they lack reciprocity and have low density (in weak-tie networks, low-reciprocity members mostly provide one-time support), and the responsibilities of members may be dispersed. Therefore, the network built around esteem and network support was not strongly connected, which may have led to the bar’s lack of esteem and network support. In addition, tangible support was less frequently found in all three stages, possibly due to the anonymity and uncertainty of information provided on the “Baidu COVID-19bar,” which makes it difficult for users to provide substantial help to others.

The results show that the social support the seekers obtained from the posts depended on the seeking strategy used. In general, the relationship between the two was not affected by the different stages of COVID-19—the conclusions are the same regardless of stage or in looking at the situation in its entirety. Asking for support was more likely to elicit informational support, and disclosing directly was more likely to elicit emotional support. Asking for support is a strategy for directly expressing individual needs, which is reflected in “Baidu COVID-19bar.” On the one hand, individuals did not seek help blindly or randomly, but rather sought reliable answers through “Baidu COVID-19bar” that gathers a large number of people who have encountered similar difficulties. On the other hand, asking for support strategy in the “Baidu COVID-19bar” usually takes the form of direct questions which represents clear requests. A two-way interaction process takes place between support seekers and potential support providers, that is, seekers formulate specific strategies, and providers evaluate the essence of the problem and their availability (Derlega et al., 2003). Therefore, people using asking for support promotes providers to understand seekers’ difficulties easily and provide specific information based on their needs in a short time, which is profit from its direct way of presenting individual needs and concise expression of their needs. Unlike asking for support, disclosing directly may or may not entail outwardly asking for help, instead users are straightforward about the source of their distress (Buehler et al., 2019). These emotional distress cues can signal both a state of need and a willingness to share one’s troubles and feelings with other trusted people (Cutrona et al., 1990). Disclosing directly strategy in “Baidu COVID-19bar” usually describes the individual’s experience of changes in symptoms, health examination results, and emotions such as anxiety and panic. Therefore, it could be speculated that although disclosing directly did not always explicitly state a need, it instead conveyed the psychological crisis and alluded to the user’s needs by explaining the events or external environment that caused their distress. Correspondingly, providers may fear that they may provide misinformation and may mislead other users. Also, the inexplicit of seekers’ information prevents them from providing informational support. Instead, they are inclined to comfort and encourage users to ease their negative emotions.

Individualistic freedom and a sense of control through individual behavior are more valued in Western individualist cultures, while social adaptation and tolerance of others are more emphasized in Eastern collectivist cultures (Markus and Schwartz, 2010). Previous studies have shown that people tend to seek help in a direct way more frequently than in an indirect way. For example, Maestre et al. (2018) found that direct support seeking was the most common strategy in online HIV health communities; Andalibi et al. (2016) similarly found that direct support seeking was more prevalent than indirect support seeking. Influenced by cultural differences in the importance of personal control and social accommodation, choosing to disclose problems and sharing personal matters is viewed as less appropriate in Eastern cultures than in Western cultures. Such disclosure implies a demand for help from others, which may disrupt harmonious relationships (Ishii et al., 2017). Therefore, in offline life, people in collectivist cultures disclose less information about themselves and are less inclined to seek help from others. However, Chinese online communities show some differences. Although not considered in terms of direct or indirect as the type of seeking strategy, asking for support and disclosing directly, which were the most common types in the “Baidu COVID-19bar,” both entailed relatively direct and explicit in communication. Therefore, whether in Western individualistic culture or Eastern collectivistic culture, people in online communities tend to seek support by means of explaining stressors directly. People can hide their identity in online communities, which may provide a channel for people in collectivistic cultures to seek help and close the gap between seeking and providing social support against different the cultural backgrounds of the East and the West.

## Conclusion and Limitations

The theoretical implication of this study is to analyse individual’s support-seeking strategies and the social support provided, as well as the relationship between the two across different stages and during the initial period of the COVID-19 pandemic. From the analysis, it was found that in all stages of the initial period of COVID-19 pandemic, informational support and emotional support were the primary social support forms offered by users in the online health community. This may have been driven by the characteristics of the online health community as well as people’s panic and uncertainty caused by the pandemic. What’s more, this study analyses support-seeking strategies and the social support provided from a micro perspective in COVID-19 pandemic, which can serve as a basis for similar studies and expand the field of research on social support. Unlike previous researches on social support under the individualist context in the West, this study enriches research findings on the characteristics of support-seeking strategies and social support, as well as the relationships between the two, provided under the collectivist context. As for the practical implications, the results of this study are of practical significance for public policy and intervention strategies. In public health emergencies, people experience anxiety, panic, and other psychological issues. Because they can strengthen their contact with others through the Internet, people can seek social support to reduce their anxiety during pandemics. Moreover, mental health organizations and all sectors of society should consider providing social support to people from online health communities, such as by developing online social support projects to meet the public’s need for social contact during public health emergencies.

Some limitations of this study are of concern, namely that posts deleted by posters or the administrators of the “Baidu COVID-19bar” will not be considered, which may undermine the integrity of the data. What’s more, this study only focuses on one online community. Thus, it provides a direction for future research, that is subsequent research could explore social support and social support seeking behaviors in a variety of online communities, as well as a comparative analysis of social support provision and seeking behaviors in online communities in different cultural contexts in the East and the West.

## Data Availability Statement

The original contributions presented in the study are included in the article/supplementary material, further inquiries can be directed to the corresponding author.

## Ethics Statement

Ethical review and approval was not required for the study on human participants in accordance with the local legislation and institutional requirements. Written informed consent for participation was not required for this study in accordance with the national legislation and the institutional requirements.

## Author Contributions

YL designed the study and performed the data analysis and wrote the article. YZ and YX coded all the original post and reply post, performed the data analysis and wrote the article. All authors contributed to this study and approved the submitted version.

## Funding

This study was supported by the Fundamental Research Funds for the Central Universities of China (Nos. 2020CDJSK07PT17, 2020CDSKXYXW008, and 2021CDSKXYXW008).

## Conflict of Interest

The authors declare that the research was conducted in the absence of any commercial or financial relationships that could be construed as a potential conflict of interest.

## Publisher’s Note

All claims expressed in this article are solely those of the authors and do not necessarily represent those of their affiliated organizations, or those of the publisher, the editors and the reviewers. Any product that may be evaluated in this article, or claim that may be made by its manufacturer, is not guaranteed or endorsed by the publisher.

## References

[ref1] Andalibi N. Haimson O. L. De Choudhury M. Forte A. (2016). Understanding Social Media Disclosures of Sexual Abuse through the Lenses of Support Seeking and Anonymity. Proceedings of the CHI Conference on Human Factors in Computing Systems, San Jose, CA, USA.

[ref2] Baidu COVID-19 bar. (2020, January 20). Retrieved March 5, 2021, fromhttps://tieba.baidu.com/f?kw=%E6%96%B0%E 5%9E%8B%E5%86%A0%E7%8A%B6%E7%97%85%E6%AF% 92&ie=utf-8.

[ref3] Barbee A. P. Cunningham M. R. (1995). An experimental approach to social support communications: interactive coping in close relationships. Ann. Int. Commun. Assoc. 18, 381–413. doi: 10.1080/23808985.1995.11678921

[ref4] Barbee A. P. Rowatt T. L. Cunningham M. R. (1998). “When a friend is in need: feelings about seeking, giving, and receiving social support,” in Handbook of Communication and Emotion: Research, Theory, Applications, and Contexts. eds. Anderson P. A. Guerrero L. K. (San Diego, CA: Academic Press), 281–301.

[ref5] Bokszczanin A. (2012). Social support provided by adolescents following a disaster and perceived social support, sense of community at school, and proactive coping. Anxiety, Stress and Coping 25, 575–592. doi: 10.1080/10615806.2011.622374, PMID: 21995730

[ref7] Brashers D. E. Neidig J. L. Goldsmith D. J. (2004). Social support and the management of uncertainty for people living with HIV or AIDS. Health Commun. 16, 305–331. doi: 10.1207/S15327027HC1603_3, PMID: 15265753

[ref6] Buehler E. M. Crowley J. L. Peterson A. M. High A. C. (2019). Broadcasting for help: a typology of support-seeking strategies on Facebook. New Media Soc. 21, 2566–2588. doi: 10.1177/1461444819853821

[ref8] Cutrona C. E. Suhr J. A. (1992). Controllability of stressful events and satisfaction with spouse support behaviors. Commun. Res. 19, 154–174. doi: 10.1177/009365092019002002

[ref9] Cutrona C. E. Suhr J. A. MacFarlane R. (1990). “Interpersonal transactions and the psychological sense of support,” in Personal Relationships and Social Support. ed. Duck S. (London: Sage), 30–45.

[ref10] Derlega V. J. Winstead B. A. Oldfield E. C. Barbee A. P. (2003). Close relationships and social support in coping with HIV: a test of sensitive interaction systems theory. AIDS Behav. 7, 119–129. doi: 10.1023/A:1023990107075, PMID: 14586197

[ref11] Dutton J. E. (1986). The processing of crisis and non-crisis strategic issues. J. Manag. Stud. 23, 501–517. doi: 10.1111/j.1467-6486.1986.tb00434.x

[ref12] Fink S. (1986). Crisis Management: Planning for the Inevitable. New York: AMACOM.

[ref13] Ford L. A. Babrow A. S. Stohl C. (1996). Social support messages and the management of uncertainty in the experience of breast cancer: An application of problematic integration theory. Commun. Monogr. 63, 189–207. doi: 10.1080/03637759609376389

[ref14] Goldsmith D. J. (2004). Communicating Social Support. New York: Cambridge University Press.

[ref15] Hawkley L. C. Cacioppo J. T. (2010). Loneliness matters: A theoretical and empirical review of consequences and mechanisms. Ann. Behav. Med. 40, 218–227. doi: 10.1007/s12160-010-9210-8, PMID: 20652462 PMC3874845

[ref16] Hether H. J. Murphy S. T. Valente T. W. (2016). A social network analysis of supportive interactions on prenatal sites. Digital Health 2, 205520761662870–205520761662812. doi: 10.1177/2055207616628700 PMC6001212 29942549

[ref17] Huh J. (2015). Clinical Questions in Online Health Communities: The Case of “See your Doctor” Threads. Proceedings of the 18th ACM Conference on Computer Supported Cooperative Work & Social Computing. Vancouver, Canada. 10.1145/2675133.2675259 PMC4488233 26146665

[ref18] Ishii K. Mojaverian T. Masuno K. Kim H. S. (2017). Cultural differences in motivation for seeking social support and the emotional consequences of receiving support: The role of influence and adjustment goals. J. Cross-Cult. Psychol. 48, 1442–1456. doi: 10.1177/0022022117731091

[ref20] Kanekar A. Sharma M. (2020). Covid-19 and mental well-being: guidance on the application of behavioral and positive well-being strategies. Healthcare 8, 336–342. doi: 10.3390/healthcare8030336, PMID: 32932613 PMC7551187

[ref21] Kleiman E. M. Yeager A. L. Grove J. L. Kellerman J. K. Kim J. S. (2020). The real-time mental health impact of the covid-19 pandemic on college students: an ecological momentary assessment study (preprint). JMIR Mental Health 7:e24815. doi: 10.2196/24815, PMID: 33207308 PMC7744138

[ref22] Ko H. C. Wang L. L. Xu Y. T. (2013). Understanding the different types of social support offered by audience to a-list diary-like and informative bloggers. Cyberpsychol. Behav. Soc. Netw. 16, 194–199. doi: 10.1089/cyber.2012.0297, PMID: 23363225 PMC3603495

[ref23] Lee C. S. (2021). Online fraud victimization in China: A case study of Baidu Tieba. Vict. Offenders 16, 343–362. doi: 10.1080/15564886.2020.1838372

[ref24] Lin N. Simeone R. S. Ensel W. M. Wen K. (1979). Social support, stressful life events, and illness: a model and an empirical test. J. Health Soc. Behav. 20, 108–119. doi: 10.2307/2136433, PMID: 479524

[ref25] MacGeorge E. L. Feng B. Burleson B. R. (2011). “Supportive communication” in The SAGE Handbook of Interpersonal Communication. eds. Knapp M. L. Daly J. A. (SAGE: Thousand Oaks, CA), 330.

[ref26] Maestre J. F. Herring S. C. Min A. Connelly C. L. Shih P. C. (2018). Where and how to look for help matters: analysis of support exchange in online health communities for people living with HIV. Information 9, 259. doi: 10.3390/info9100259

[ref27] Markus H. R. Schwartz B. (2010). Does choice mean freedom and well-being? J. Consum. Res. 37, 344–355. doi: 10.1086/651242

[ref28] Nambisan P. (2011). Information seeking and social support in online health communities: impact on patients’ perceived empathy. J. Am. Med. Inform. Assoc. 18, 298–304. doi: 10.1136/amiajnl-2010-000058, PMID: 21486888 PMC3078657

[ref29] National Health Commission of the People’s Republic of China. (2020). National Health Commission there were no new confirmed cases in mainland China Available at:18. https://baijiahao.baidu.com/s?id=1661576455862897097&wfr= spider&for=pc (Accessed on March 18, 2020).

[ref31] Oh H. J. LaRose R. (2016). Impression management concerns and support-seeking behavior on social network sites. Comput. Hum. Behav. 57, 38–47. doi: 10.1016/j.chb.2015.12.005

[ref32] Panteli N. Sims J. (2010). Developing Virtual Medical Communities. Proceedings of the IADIS International Conference on e-Health. ed. Macedo M. Freiburg, Germany.

[ref34] Qian Y. Gui W. Ma F. Dong Q. (2021). Exploring features of social support in a Chinese online smoking cessation community: A multidimensional content analysis of user interaction data. Health Informatics J. 27, 146045822110214. doi: 10.1177/14604582211021472 34082598

[ref36] Sims J. (2011). Communities of Practice, Telemedicine and Collaborative Technologies in Palliative Care: A Work in Progress. Proceedings of the IADIS International Conference on Information Systems. eds. Nunes M. B. Isaías P. Powell P. Avila, Spain.

[ref37] Tanis M. (2008). Health-related online forums: What’s the big attraction. J. Health Commun. 13, 698–714. doi: 10.1080/10810730802415316, PMID: 18958781

[ref38] The State Council Information Office of the People's Republic of China. (2020). White paper on Fighting Covid-19 China in Action. Available at: http://www.scio.gov.cn/ztk/dtzt/42313/43142/index.htm.

[ref39] Taylor S. E. Broffman J. I. (2011). Psychosocial resources: functions, origins, and links to mental and physical health. Adv. Exp. Soc. Psychol. 44, 1–57. doi: 10.1016/B978-0-12-385522-0.00001-9

[ref40] Wang G. Zhang W. Zeng R. X. (2019). WeChat use intensity and social support: The moderating effect of motivators for WeChat use. Comput. Hum. Behav. 91, 244–251. doi: 10.1016/j.chb.2018.10.010

[ref41] Williams S. L. Mickelson K. D. (2008). A paradox of support seeking and rejection among the stigmatized. Pers. Relat. 15, 493–509. doi: 10.1111/j.1475-6811.2008.00212.x

[ref42] World Health Organization (WHO) (2020). WHO declared the COVID-19 outbreak a public health emergency of international concern. Available at: https://www.euro.who.int/en/health-topics/health-emergencies/international-health-regulations/news/news/2020/2/2019-ncov-outbreak-is-an-emergency-of-international-concern#:~:text=The%20WHO%20Director-General%2C%20Dr%20Tedros%20Adhanom%20Ghebreyesus%2C%20declared, Health%20Regulations%20%28IHR2920came%20into%20force%20in%202005.

[ref43] Wright K. B. Cai X. M. Fisher C. Rising C. J. Burke-Garcia A. Afanaseva D. (2021). A content analysis of social support messages about environmental breast cancer risk within blogs for mothers. Health Commun. 36, 1796–1804. doi: 10.1080/10410236.2020.1800241 32744079 PMC7855537

[ref44] Xu J. Ou J. Luo S. Wang Z. Chang E. Novak C.. (2020). Perceived social support protects lonely people against covid-19 anxiety: a three-wave longitudinal study in China. Front. Psychol. 11, 1–12. doi: 10.3389/fpsyg.2020.566965 33240152 PMC7677578

[ref45] Yao Z. Tang P. Fan J. R. Luan J. (2021). Influence of online social support on the public’s belief in overcoming covid-19. Inf. Process. Manag. 58:102583. doi: 10.1016/j.ipm.2021.102583, PMID: 33746338 PMC7959279

[ref46] Ye J. W. Yeung D. Y. Liu E. S. C. Rochelle T. L. (2019). Sequential mediating effects of provided and received social support on trait emotional intelligence and subjective happiness: A longitudinal examination in Hong Kong Chinese university students. Int. J. Psychol. 54, 478–486. doi: 10.1002/ijop.12484 29611619

[ref19] Yip J. W. C. (2020). Evaluating the communication of online social support: A mixed-methods analysis of structure and content. Health Commun. 35, 1210–1218. doi: 10.1080/10410236.2019.1623643 31154856

